# *In vitro* interaction between Ecteinascidin 743 (ET-743) and radiation, in relation to its cell cycle effects

**DOI:** 10.1038/sj.bjc.6601431

**Published:** 2003-12-09

**Authors:** C Simoens, A E C Korst, C M J De Pooter, H A J Lambrechts, G G O Pattyn, G T Faircloth, F Lardon, J B Vermorken

**Affiliations:** 1 Laboratory of Cancer Research and Clinical Oncology, Department of Medical Oncology, University of Antwerp (UIA/UZA), Universiteitsplein 1, B-2610 Wilrijk, Antwerp, Belgium; 2 Department of Radiotherapy, St Augustinus Hospital, Oosterveldlaan 24, B-2610 Wilrijk, Antwerp, Belgium; 3 Preclinical Drug Development, PharmaMar USA, Inc., 320 Putnam Avenue, Cambridge, MA 02139, USA

**Keywords:** ET-743, radiation, radiosensitisation, cell cycle

## Abstract

Ecteinascidin 743 (ET-743) is a new marine-derived agent with promising activity against a number of solid tumours. In four human tumour cell lines, the interaction between ET-743 and radiation was investigated in relation to the effects of ET-743 on the cell cycle, *in vitro*. Cell survival was measured based on quantitative staining of cellular protein by sulforhodamine B. A 24 h treatment with ET-743 before radiation resulted in a moderate increase in radiosensitivity in three out of four cell lines. Dose enhancement factors ⩾1.8 were observed for concentrations resulting in 52, 46 and 30% cell kill in ECV304, H292 and CAL-27, respectively, whereas in A549 no radiosensitisation was observed (no significant increase in radiosensitivity). According to the combination index analysis, synergism was observed only in ECV304 and CAL-27 cells. A 24 h incubation with ET-743 resulted in a concentration-dependent G2/M block, which might explain the moderate radiosensitising effects in ECV304 and H292. The lack of radiosensitisation in A549 might be due to the S phase delay preceding the G2/M block at the moment of radiation, which only occurred in this cell line. In conclusion, ET-743 has moderate cell line-dependent radiosensitising properties; however, only when cytotoxic concentrations of ET-743 are used. In one of the four cell lines tested, no radiosensitisation was observed.

Natural products from plants and marine organisms are potential rich sources of new anticancer agents with possible new mechanisms of action. Plant-derived agents such as paclitaxel and docetaxel have already proved to be important drugs for the treatment of cancer, and an increasing number of substances originating from marine organisms are being investigated in preclinical studies and clinical trials. Among the marine anticancer agents, the ecteinascidins (ETs) merit special consideration. These colonial tunicates are produced by *Ecteinascidia turbinata* and grow preferentially on mangrove roots. Ecteinascidin 743 (ET-743) (Yondelis™) is the most promising compound, based on its cytotoxicity and its abundance in the tunicate (Jimeno *et al*, 1996).

Ecteinascidin 743 is a tetrahydroisoquinoline agent (Figure 1

**Figure 1:**
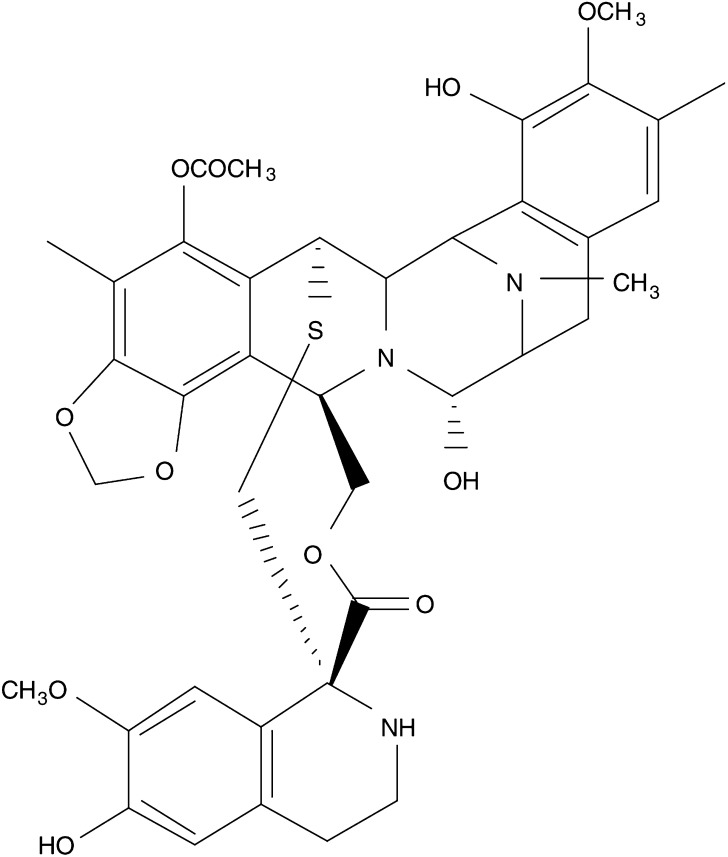
Chemical structure of ET-743.

) that binds the minor groove of DNA and forms covalent adducts at the N2 of guanine (Pommier *et al*, 1996; Zewail Foote and Hurley, 1999). A unique feature among DNA-interactive agents that occupy the minor groove is that ET-743 bends DNA towards the major groove (Zewail Foote and Hurley, 1999; Garcia Nieto *et al*, 2000). Also, the sequence specificity of the interaction between ET-743 and DNA is revealed, indicating that DNA duplexes containing GC-rich sites (5′-PuGC-3′ and 5′-PyGG-3′) are putative alkylation sites. The implications of the study by Moore *et al* (1998) are that while units A and B of ET-743 recognise and bind to DNA duplexes, unit C is directed out of the minor groove and might directly interact with transcription factors (Pommier *et al*, 1996). Ecteinascidin 743 inhibits promotor-specific induction of several important genes, including p21^WAF−1/CIP−1^, c-Jun, c-Fos, MDR1 and others. This compound is a novel, potent and general inhibitor of activated but not constitutive transcription (Friedman *et al*, 2002). Ecteinascidin 743 also interferes with the DNA repair pathway, namely the transcription-coupled nucleotide excision repair (TC-NER) pathway (Damia *et al*, 2001; Takebayashi *et al*, 2001; Zewail Foote *et al*, 2001), which might be important when ET-743 is combined with other DNA-damaging agents. Ecteinascidin 743 also causes perturbation of the cell cycle with a delay in the progress through the S phase, finally resulting in a G2/M phase block (Erba *et al*, 2001; Li *et al*, 2001).

*In vitro* studies with ET-743 have demonstrated activity at nanomolar concentrations, with a continuous 24 h exposure being more active than a single 1 h exposure (Ghielmini *et al*, 1998; Izbicka *et al*, 1998). Ecteinascidin 743 has proven to be a potent drug against a number of solid tumour cell lines and human tumour xenografts, including soft-tissue sarcoma, melanoma, NSCLC, ovarian, breast, prostate and renal cancer (Jimeno *et al*, 1996; Hendriks *et al*, 1999; Li *et al*, 2001).

Ecteinascidin 743 is under active phase II (III) development in different tumour types. Phase II studies both in the US and Europe have indicated that ET-743 is an active agent for the treatment of patients with soft-tissue sarcoma (Le Cesne *et al*, 2001; Demetri, 2002). A pooled analysis of pivotal phase II trials in pretreated advanced soft-tissue sarcomas demonstrated an objective response rate of 9.4% and an additional minor response rate of 8.7%, a 1-year survival of 40% and a progression-free survival at 6 months of 27% (Le Cesne *et al*, 2001). In addition, early data in ovarian cancer patients relapsing (⩾6 months) after platinum–taxane therapy indicated a 47% response rate with ET-743 in 17 patients (Parma *et al*, 2003). The synergistic effect observed in preclinical models and these clinical data will prompt evaluation of ET-743 in combination with cisplatin or doxorubicin in ovarian cancer patients in the near future. Other tumour types in which ET-743 is presently being explored are breast cancer and endometrial cancer (Zelek *et al*, 2000).

In recent years, combined treatments of chemotherapy and radiotherapy have been investigated extensively in clinical studies, revealing promising applications for a variety of malignancies such as NSCLC, head and neck, oesophageal and cervical cancer (Pignon *et al*, 2000; Geh, 2002; Kim *et al*, 2002; Lukka *et al*, 2002; Rose, 2002). This may result from an improvement in systemic and local tumour control, and from direct interactions between cytotoxic agents and radiation, leading to increased antitumour activity. These interactions may be caused by DNA repair inhibition, cell cycle redistribution or altered cytokinesis or apoptosis (Hennequin and Favaudon, 2002). For new chemotherapeutic agents, it is therefore important to investigate possible interactions with radiotherapy *in vitro*, before applying concomitant chemoradiotherapy in the clinic. However, one should keep in mind that radiosensitising effects do not by definition lead to an improved therapeutic index. The selectivity of the interaction between the cytotoxic agents and radiotherapy is a key issue for improved clinical outcome. Although ET-743 shows a peculiar pattern of selectivity in cells with different defects in their DNA-repair pathways (D'Incalci *et al*, 2002), which suggests normal cells to be less affected by ET-743, *in vivo* studies should always be used to verify an improved therapeutic index.

The aim of the present study was to investigate the interaction between the novel agent ET-743 and radiotherapy *in vitro*. Since cell cycle perturbations frequently play a role in interactions between chemotherapy and radiation, the effect of ET-743 on the cell cycle was also investigated.

## MATERIALS AND METHODS

### Cell lines

Four different human tumour cell lines have been used: ECV304, an epidermoid bladder cancer cell line; H292, a mucoepidermoid lung cancer cell line; A549, a squamous lung cancer cell line and CAL-27, a squamous cell carcinoma cell line of the tongue. ECV304 cells were grown in Medium 199 (Bio Whittaker, Verviers, Belgium) supplemented with 10% fetal calf serum (Invitrogen, Merelbeke, Belgium). H292 and A549 cells were cultured in RPMI-1640 medium, supplemented with 2 mM glutamine, 1 mM sodium pyruvate (Invitrogen) and 10% fetal calf serum. CAL-27 cells were grown in Dulbecco's modified Eagle's medium (DMEM) medium, supplemented with 2 mM glutamine and 10% fetal calf serum. No antibiotics were added to the media. The cultures were maintained in exponential growth at 37°C in a humidified 5% CO_2_ atmosphere.

### Ecteinascidin 743

Ecteinascidin 743 was kindly provided by Dr Glynn T Faircloth, PharmaMar USA, Inc., Cambridge, MA, USA. Ecteinascidin 743 was dissolved in dimethyl sulphoxide (DMSO) to create a concentrated stock solution of 660 *μ*M and stored in aliquots at −20°C. Aliquots were thawed for each new experiment. Before use, the stock solution was diluted in phosphate-buffered saline (PBS: 0.03 M KCl, 1.37 M NaCl, 0.01 M KH_2_PO_4_, 0.06 M Na_2_HPO_4_) to the desired concentrations.

### Cell survival experiments

Cells were harvested from exponential phase cultures by trypsinisation, counted and plated at optimal seeding densities in 48-well plates, to assure exponential growth during the experiments. Cell densities were about 100 cells well^−1^ for all the cell lines tested. Cells were treated, after a 24 h recovery period, in two treatment schedules: 24 h incubation with ET-743 followed by radiation and 24 h incubation with ET-743 just after radiation (Cobalt-60 *γ*-rays, 0–8 Gy, room temperature). Cells were washed with a drug-free medium after 24 h incubation with the drug, and maintained at 37°C. At 7 or 8 days (about six doubling times) after the radiation treatment, cell survival was determined by the sulforhodamine B (SRB) assay. Each concentration was tested six times within the same experiment. All experiments were performed at least three times.

The SRB test is a suitable test system for *in vitro* radiosensitivity testing, which has shown to be comparable in outcome with the clonogenic assay, when cells are allowed to undergo at least six doubling times after irradiation (Pauwels *et al*, 2003). Therefore, in our experiments, ECV304, H292 and A549 cells were incubated for 7 days and CAL-27 cells for 8 days after radiation, before survival assessments by the SRB assay. The SRB assay was performed according to the method of Skehan *et al* (1990) and Papazisis *et al* (1997), with minor modifications. In brief, the culture medium was aspirated prior to fixating the cells by the addition of 200 *μ*l 10% cold trichloroacetic acid. After 1 h incubation at 4°C, cells were washed five times with deionised water. Cells were then stained with 200 *μ*l 0.1% SRB (ICN, Asse, Belgium), dissolved in 1% acetic acid for at least 15 min and washed four times with 1% acetic acid to remove unbound stain. The plates were left to dry at room temperature and the bound protein stain was solubilised with 200 *μ*l 10 mM unbuffered TRIS base (tris(hydroxymethyl) aminomethane) and transferred onto 96-well plates for reading the optical density at 540 nm (Biorad 550 microplate reader, Nazareth, Belgium).

### Cell cycle experiments

Cells from exponential phase cultures were trypsinised and plated in 6-well plates. In order to assure exponential growth during the experiment, seeding densities were 20 000 cells well^−1^ for ECV304 and A549, and 25 000 cells well^−1^ for H292. Following plating and a 24 h recovery period, cells were incubated with different concentrations of ET-743 near the IC_50_. After a 24 or 48 h incubation period, cell cycle analysis was performed immediately, 4 or 24 h later, by flow cytometry.

DNA was stained according to the Vindelov method, after trypsinisation (Vindelov *et al*, 1983). In brief, cells were resuspended in 100 *μ*l PBS and after addition of 100 *μ*l solution A (trypsin), the cells were incubated for 20 min at room temperature. Then, 75 *μ*l solution B (trypsin inhibitor spermine and ribonuclease A) was added and after 10 min incubation at room temperature, 75 *μ*l solution C (propidium iodide) was added for at least 30 min at 4°C. Samples were analysed in a FACScan flow cytometer (Becton-Dickinson, San José, CA, USA).

### Data analysis and statistics

#### Cell survival experiments

The survival rates were calculated by: mean optical density (OD) of treated cells/mean OD of untreated cells × 100%. Radiation dose–survival curves were fitted according to the linear-quadratic model: survival=exp(−*αD*-*βD*^2^), using WinNonlin (Pharsight, Palo Alto, CA, USA). The radiation dose–survival curves were corrected for the cytotoxic effect of ET-743 alone (the curves were displaced in a vertical direction, so all dose–survival curves start at 100% survival). From the dose–survival curves, the ID_50_ was calculated, which is the radiation dose causing 50% growth inhibition. A two-sample *t*-test was used to investigate significant differences between ID_50_ values. The results are expressed as mean±s.d.

Radiosensitisation was expressed by the dose enhancement factor (DEF): ID_50_ of the untreated cells/ID_50_ of the cells treated with ET-743.

Possible synergism was determined by the calculation of the combination index (CI) by the Chou and Talalay (1984) equation, using CalcuSyn (Biosoft, Cambridge, UK), which can also be used for chemoradiation combinations (Leonard *et al*, 1996). The CI takes into account both the potency (IC_50_ or *D*_*m*_) and the shape of the dose–survival curve (*m* value). The general equation for the classic isobologram is given by:


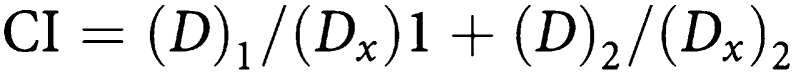


where (*D*_*x*_)_1_ and (*D*_*x*_)_2_ are the doses (or concentrations) for *D*_1_ (ET-743) and *D*_2_ (radiation) alone that give *x*% inhibition, whereas (*D*)_1_ and (*D*)_2_ are the doses of ET-743 and radiation in combination that also inhibit *x*% (i.e. isoeffect).

The (*D*_*x*_)_1_ or (*D*_*x*_)_2_ (for ET-743 and radiation) are calculated by the formula:


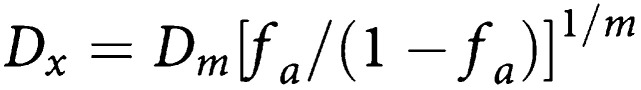


where *D*_*m*_ is the dose required to produce absorbance readings 50% lower than those of nontreated wells (IC_50_ or ID_50_), *f*_a_ is the fraction affected and *m* is the slope of the median-effect plot. The CI values obtained from the classic (mutually exclusive) isobologram calculations are given. In short, 1.10>CI>0.90, 0.90>CI>0.85, 0.85>CI>0.70 and 0.70>CI>0.30 indicates a nearly additive effect, slight synergism, moderate synergism and synergism, respectively.

#### Cell cycle experiments

Flow cytometric data were analysed using Cell Quest (Becton Dickinson). A one-way analysis of variance (ANOVA), followed by a Bonferroni adjustment of the *P*-value, was used to investigate the significance of the differences between the percentages of cells in the different cell cycle phases after treatment with ET-743 *vs* the untreated cells.

## RESULTS

The four cell lines used in this study were almost equally sensitive to the cytotoxic effect of ET-743, with mean IC_50_ values of 1.6±0.6, 1.1±0.5, 0.9±0.4 and 1.5±0.5 nM for ECV304, H292, CAL-27 and A549, respectively.

### Chemoradiation experiments

Figure 2

**Figure 2:**
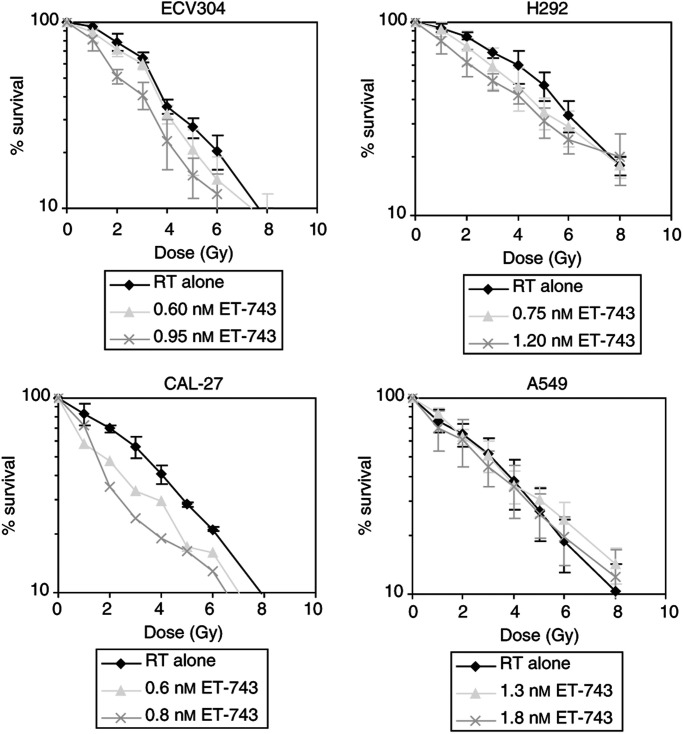
Survival *vs* radiation dose curves of four human tumour cell lines treated with radiation alone *vs* different concentrations of ET-743 for 24 h prior to radiation. (Survival curves were corrected for the cytotoxic effect of ET-743 itself.) RT=radiotherapy; Gy=gray.

illustrates dose–response curves of the four cell lines when treated with the combination of ET-743 and radiation. The results of cells treated with two different concentrations of ET-743 for 24 h before irradiation are shown (other concentrations are also tested and an overview of the complete results is shown in Figure 3

**Figure 3:**
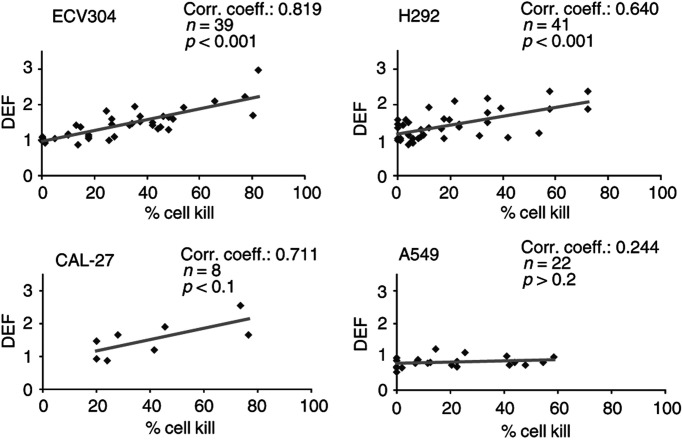
Correlation between percentage cell kill caused by ET-743 alone and the DEF in four human tumour cell lines. n=number of values.

). Radiation doses ranged from 0 to 8 Gy.

In ECV304, H292 and CAL-27, a significant decrease in ID_50_ values of the ET-743-treated cells compared with the untreated cells was observed (*p*<0.001). Pretreatment with ET-743 therefore seemed to increase the radiosensitivity of ECV304, H292 and CAL-27 cells. On the contrary, no radiosensitising effect was observed in A549.

An increase in radiosensitivity can be described by the DEF. This DEF seemed to correlate with the cytotoxic effect of ET-743 alone. In Figure 3, the correlation between the percentage cell kill caused by ET-743 alone and the DEF is shown for the four cell lines. In ECV304, H292 and CAL-27, more toxic concentrations of ET-743 resulted in a higher DEF. In A549, no correlation was found; even toxic concentrations of ET-743 did not result in a radiosensitising effect (DEF=1.29 for concentrations around IC_50_, without a significant decrease in ID_50_). In the other three cell lines, rather toxic concentrations were needed to obtain a clear radiosensitising effect: DEFs ⩾1.8 were observed for concentrations resulting in 52, 46 and 30% cell kill in ECV304, H292 and CAL-27, respectively.

To investigate whether these concentrations result in a synergistic effect between ET-743 and radiation, the CI was calculated. In ECV304, moderate synergism was observed for concentrations around IC_80_, with a CI value of 0.74 (DEF=2.03). For concentrations below the IC_80_, additivity was found. In H292, the calculation of the CI resulted in a value between 0.90 and 1.10 for concentrations between IC_40_ and IC_80_, so only a nearly additive effect. In CAL-27, concentrations of ET-743 between IC_30_ and IC_50_ already gave a CI value of 0.82 (DEF=1.88), resulting in moderate synergism. Concentrations around the IC_50_ resulted in a clear synergistic effect, with a CI value of 0.65 (DEF=2.23). In A549 cells, no synergism was observed (CI=1.08).

**Figure 4:**
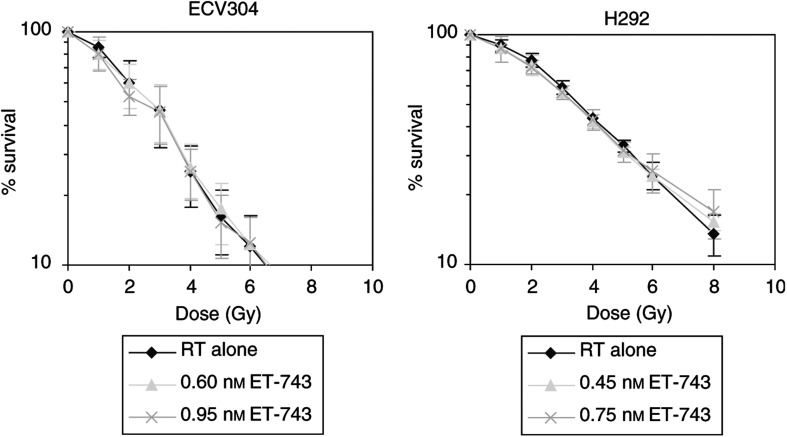
Survival *vs* radiation dose curves of ECV304 and H292 cells, treated with radiation alone *vs* different concentrations of ET-743 for 24 h after radiation. (Survival curves were corrected for the cytotoxic effect of ET-743 itself.) RT=radiotherapy; Gy=gray.

In the other treatment schedule, 24 h incubation with ET-743 immediately after radiation, no radiosensitising effect was observed in the four cell lines tested. Figure 4

gives an illustration of this for ECV304 and H292 cells. The DEF was 1.03 for 0.95 nM ET-743 in ECV304; and 1.07 for 0.75 nM ET-743 in H292.

### Cell cycle experiments

Treatment with ET-743 caused an accumulation of cells in the G2/M phase of the cell cycle. This cell cycle effect is concentration dependent in the three cell lines tested. In ECV304 cells, the percentage of cells in the G2/M phase increased from 14.2±0.9% in the untreated cells, to 31.9±2.9% after 48 h incubation with 2 nM ET-743 and to 37.8±1.8% after 48 h incubation with 3 nM ET-743. In A549 cells, there was an increase from 15.1±0.8% in untreated cells, to 43.0±7.5% after 48 h with 0.8 nM ET-743 and to 67.4±3.3% after 48 h with 1.8 nM ET-743.

In Figure 5

**Figure 5:**
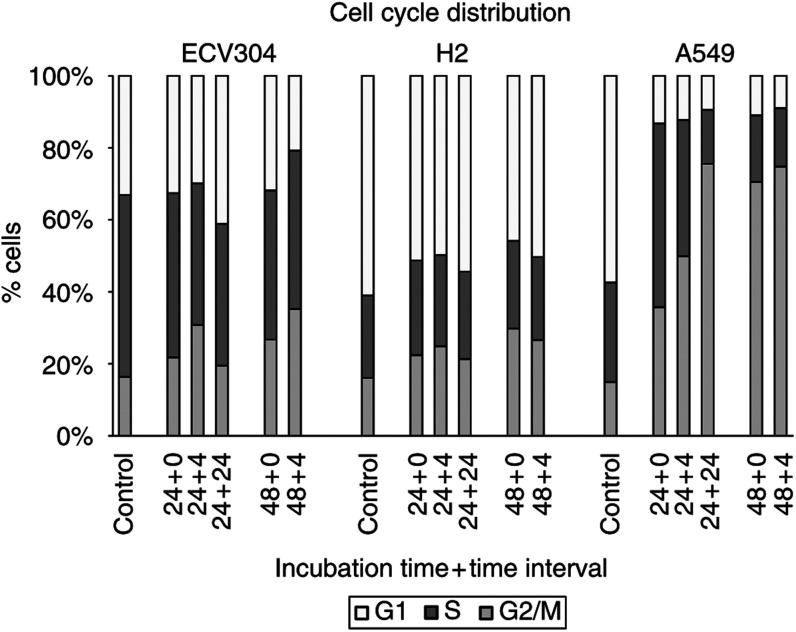
Cell cycle distribution of ECV304, H292 and A549 after incubation with ET-743 using different incubation times (24, 48 h) and time intervals (0, 4, 24 h). The following concentrations of ET-743 were used: ECV304: 1.5 nM ET-743; H292: 0.75 nM ET-743; A549: 1.8 nM ET-743.

and Table 1

**Table 1:** Cell cycle distribution of ECV304, H292 and A549 after incubation with ET-743 using different incubation times (24, 48 h) and time intervals (0, 4, 24 h)

	**Cell cycle phases (%)**		
**Cell line – conc. ET-743**	**G1**	**S**	**G2/M**
ECV – 1.5 nM			
Control	33.1±4.2	50.6±5.4	16.3±2.1
			
24 h	32.6±5.2	45.6±6.1	21.7±3.9^a^
24+4 h	29.9±5.5	39.4±4.7^b^	30.8±4.7^b^
24+24 h	41.1±6.1	39.4±8.6^a^	19.4±2.6
			
48 h	31.8±5.1	41.4±3.0^a^	26.7±4.4^b^
48+4 h	20.7±3.6^a^	43.8±4.4	35.0±4.3^b^
			
H292 – 0.75 nM			
Control	60.8±4.9	22.8±3.3	16.0±1.9
			
24 h	51.1±1.5^b^	26.2±1.1	22.3±1.9^a^
24+4 h	49.7±3.5^a^	25.3±2.7	24.8±1.9^a^
24+24 h	54.2±2.5	24.2±2.4	21.2±0.6
			
48 h	45.6±4.8^b^	24.2±3.1	29.6±5.6^b^
48+4 h	50.1±1.7^a^	22.9±2.4	26.4±3.5^b^
			
A549–1.8 nM			
Control	57.1±1.3	27.5±1.2	14.8±1.0
			
24 h	13.2±1.7^b^	50.9±5.4^b^	35.6±4.8^b^
24+4 h	12.2±3.1^b^	37.7±5.4^a^	49.6±5.1^b^
24+24 h	9.2±1.4^b^	14.6±3.1^a^	73.6±1.9^b^
			
48 h	10.5±1.3^b^	17.7±4.8^a^	67.4±3.3^b^
48+4 h	8.7±0.9^b^	15.7±2.3^a^	72.3±2.0^b^

a *p*<0.05 *vs*control.

b *p*<0.005 *vs* control.

, the cell cycle distribution data after treatment with ET-743 during 24 or 48 h, are summarised for ECV304, H292 and A549 cells. Flow cytometry was performed immediately, 4 or 24 h (not used after 48 h incubation) after treatment. The concentrations used in these cells cycle experiments are close to, or in the range of the observed IC_50_ values of the cell lines. In all the three cell lines, an increase in incubation time (48 h instead of 24 h) resulted in an increase in the amount of cells in the G2/M phase. At 4 h after a 24- or 48 h-incubation period, the percentage of cells in G2/M still increased, although less pronounced in H292. At 24 h after a 24 h-incubation period, cell cycle distributions are restored, except in A549 cells. In this cell line, 24 h after a 24 h-incubation period, cells were still accumulating in the G2/M phase. The duration of this G2/M block seemed to be concentration dependent: 24 h after 24 h incubation with 1.8 nM ET-743, the percentage of cells in G2/M phase was further increasing, while for a lower concentration of ET-743 (0.8 nM), the same effect as in ECV304 was observed (data not shown). As shown in Figure 6

**Figure 6:**
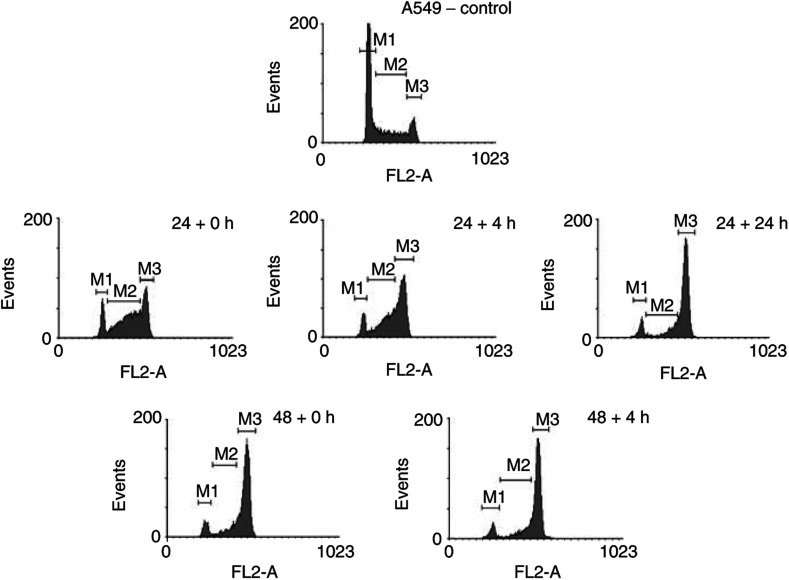
DNA histograms of A549 cells after incubation with 1.8 nM ET-743 during a 24 or 48 h incubation period and analysed by flow cytometry, 0, 4 or 24 h (not used after 48 h incubation) later. M1=G1 phase; M2=S phase; M3=G2/M phase.

, the G2/M block in A549 cells was preceded by a delay in the S phase after 24 h incubation with ET-743.

## DISCUSSION

In the current study, we investigated the interaction between ET-743 and radiation and the effects of ET-743 on the cell cycle, *in vitro*. Treatment with ET-743 during 24 h before radiation resulted in a moderate increase in radiosensitivity in three out of four cell lines. In ECV304, H292 and CAL-27, a significant decrease in ID_50_ was observed. The decrease in ID_50_ or the increase in DEF clearly correlated with the cytotoxic effect of ET-743 alone. Combination index analysis showed moderate synergism in ECV304 for quite toxic concentrations around the IC_80_. In H292 and for lower concentrations in ECV304, additivity was found. In CAL-27, synergism was already observed with concentrations around the IC_50_, whereas in A549 cells, treatment with ET-743 did not influence the radiosensitivity of the cell line. Treatment with ET-743 during 24 h after radiation instead of before did not result in any radiosensitisation.

It has long been known that radiosensitivity changes with the progression of cells through the cell cycle; while the S phase is most radioresistant, the G2/M phase is usually considered to be most radiosensitive (Terasima and Tolmach, 1963; Sinclair and Morton, 1966). Synchronisation of the cells in G2/M is expected to elicit the maximum response to radiotherapy. For a few years, this was also proposed as a rationale for the use of the taxanes in combination with radiotherapy. Pioneering studies indicated that increased radiosensitivity occurred at the time of the G2/M block. However, further studies showed a cell line-dependent radiosensitisation, which was not always associated with the G2/M block (Hennequin and Favaudon, 2002).

The influence of ET-743 on the cell cycle was investigated in ECV304, H292 and A549 cells. In all the three cell lines, a concentration-dependent G2/M block was observed after 24 h incubation with ET-743, which confirmed earlier results (Erba *et al*, 2001; Li *et al*, 2001). This G2/M block might explain the moderate radiosensitising effect in ECV304 and H292. However, since this G2/M block did not result in a radiosensitising effect in A549, this cell cycle effect is probably not the only factor for radiosensitisation. On the other hand, the lack of radiosensitisation in A549 cells might be due to the S phase delay at the moment of radiation, which precedes the G2/M block in this cell line. Immediately after 24 h incubation with ET-743, 50.9% of the cells are still in the S phase, whereas 35.6% are present in G2/M. After 48 h incubation with ET-743 or 24 h after 24 h- incubation, the G2/M block is maximal and the percentage of cells in S was reduced to 17.7 and 14.6%, respectively. This might suggest that when A549 cells would be irradiated after 48 h incubation with ET-743 or 24 h after a 24 h- incubation period, radiosensitisation might occur. When radiosensitisation indeed is associated with the G2/M block in the other cell lines, other treatment schedules might also give more radiosensitisation, such as a 4 h interval between a 24 h incubation period and radiation in ECV304.

Cell cycle effects by ET-743 are cell line-dependent, an observation that has been described previously (Erba *et al*, 2001; Li *et al*, 2001). The reason for this cell line dependency still has to be clarified.

A G2/M block was found in our study, probably caused by treatment with ET-743. However, care should be taken in assuming a direct drug-related block because dying cells that are accumulating in mitosis can also increase the G2/M fraction. However, if it were only dying cells accumulating in G2/M, instead of a drug-related block, no radiosensitising effect would be expected. However, radiosensitisation was seen in our chemoradiation experiments. To distinguish between dying cells accumulating in G2/M and a drug-related block, apoptosis *vs* cell cycle experiments can be performed.

The *in vitro* concentrations used in our study (1–2 nM) approximate to peak plasma concentrations observed with 24 h infusion of ET-743 in patients (approximately 2.3 nM) (Taamma *et al*, 2001). However, whether the observed moderate radiosensitising effect will be expected in patients, and especially whether the therapeutic index will be increased, still has to be investigated. For this reason, *in vivo* testing should be considered for further elucidation of the clinical relevance of the combination of ET-743 and radiotherapy.

In conclusion, ET-743 has moderate cell line-dependent radiosensitising properties. Radiosensitisation might be due to a G2/M block, and when this would be an important factor, radiosensitisation might be more pronounced when cells are irradiated 4–24 h after incubation with ET-743, or when the incubation period is prolonged to 48 h. However, further investigation is necessary to confirm the role of the cell cycle effects caused by ET-743 in the observed radiosensitisation.
